# Association between outdoor air pollution and semen quality: Protocol for an updated systematic review and meta-analysis

**DOI:** 10.1097/MD.0000000000015730

**Published:** 2019-05-17

**Authors:** Jianzhong Zhang, Zhonglin Cai, Bin Yang, Hongjun Li

**Affiliations:** a Department of Urology, Peking Union Medical College Hospital, Peking Union Medical College, Chinese Academy of Medical Sciences, Beijing; b Department of Urology, The Affiliated Hospital of Qingdao University, Qingdao, Shandong Province, China.

**Keywords:** air pollution, meta-analysis, sperm quality, trial sequential analysis

## Abstract

**Background:**

Various studies have explored the association between outdoor air pollution and semen quality. However, the results were still controversial. The object of the current meta-analysis is to evaluate the role of outdoor air pollution in semen quality.

**Methods:**

Databases including PubMed, Web of Science, and Embase will be searched to identify qualified studies. All qualified cross-sectional studies researching the association between outdoor air pollution and sperm parameters will be included. Relative data in participants under higher exposure and lower exposure to air pollution will be extracted by 2 investigators independently. Only participants under the highest and the lowest exposure will be enrolled if the original study contained more than 2 exposure levels. The semen volume, sperm concentration, progressive motility, total motility, and normal morphology rate will be the primary outcomes of the current study. Pooled estimates with corresponding 95% confidence intervals will be calculated to assess the specific effects of outdoor air pollution in semen quality. Moreover, trial sequential analyses will be performed to obtain a more comprehensive assessment of analyses.

**Results:**

A high-quality synthesis of the current evidence for the association between sperm parameters and outdoor air pollution will be provided.

**Conclusions:**

This meta-analysis and systematic review will generate evidence for judging whether outdoor air pollution can impair semen quality.

**PROSPERO registration number:**

PROSPERO CRD 42019126060

## 1 Introduction

Air pollution, occurs when harmful or excessive quantities of substances are released into the atmosphere, is a leading environmental health issue worldwide. Air pollutants can be in the form of gases, solid particles, or liquid droplets. The most common sources of air pollution include particulates, ozone, nitrogen dioxide, and sulfur dioxide. It may cause allergies, diseases, and even death to humans. The World Health Organization (WHO) estimated in 2014 that air pollution causes the premature death of 7 million people per year worldwide. The incidence is higher in subjects with low socioeconomic status.^[1]^ Air pollution can result in various diseases including lung diseases, cardiovascular and neurologic disorders, and infertility.^[2–5]^ Recently, various studies have focused on the effects of air pollution on male fertility.^[6,7]^

During the past decades, a large amount of studies has reported consistent degradation in human semen quality in healthy males and the WHO has lowered the normal reference range of sperm parameters based on this recognition. Various potential causes were reported associated with degradation in human semen quality, including exposure to pollutants or toxicants, polycyclic aromatic hydrocarbon, overweight, obesity, smoking, drinking, psychological stress, and several specific chronic diseases.^[6,8–11]^ Among these factors, outdoor air pollution has been the hotpot of the current related studies considering large amount of the affected populations. The biological mechanism of the effects of air pollution on sperm quality remains unclear. Several possible mechanisms were identified, including impairment of sperm DNA, disorders of hypothalamic pituitary axis and testicular spermatogenesis, and oxidative stress.

Though fundamental researches supported the negative effects of air pollution on sperm quality, several epidemiologic studies demonstrated nonsignificant or contrary results. For instance, with regard to sperm count, several studies have demonstrated that air pollution can result in significant reduction in sperm concentration^[12–15]^ and total sperm count,^[13–15]^ while the other data did not show significant decrease or even increase of these indicators. In terms of sperm motility, air pollution was reported associated with decreased progressive motility^[12,13,16–18]^ and total motility.^[13,16–19]^ But other studies did not demonstrate significant results.

Meta-analysis is a powerful tool which can provide more reliable results by pooling the results of single studies, especially in explaining controversial conclusions. The present study is designed to synthesize currently available evidences to evaluate the specific influences of air pollution on semen parameters.

## 2 Methods

The protocol has been registered on the international prospective register of systematic reviews (PROSPERO registration number: CRD 42019126060) and was strictly reported based on the Preferred reporting items for systematic review and meta-analyses protocols statement. The study was approved by the Ethics Committee of Peking Union Medical College Hospital, Beijing, China.

### 2.1 Evidence acquisition

Qualified studies will be identified by a systematic search of the following databases: PubMed, Web of Science, and Embase, updated to January 1, 2019.

MeSH words and free words with the following searching strategy will be used: (“air pollution” OR “air pollutants” OR “PM_10_” OR “PM_2.5_” OR “suspended particulates” OR “NO_x_” OR “NO” OR “NO_2_” OR “SO_2_” OR “carbon monoxide” OR “ozone” OR “particulate matter” OR “soot” OR “smog” OR “volatile organic compounds” OR “chlorofluorocarbons” OR “traffic” OR “motor vehicles”) AND (“semen quality” OR “semen volume” OR “sperm concentration” OR “sperm count” OR “sperm motility” OR “sperm morphology” OR “sperm DNA fragmentation”). In addition to electronic search original papers, reference lists of the identified original articles and reviews will be hand-searched to prevent missing any further studies. If more research data will be required, we will contact the authors to get desired information.

Enrolled studies in the current meta-analysis must meet the following criteria: English publications; longitudinal or cross-sectional studies; researches associated with the effects of outdoor air pollution on sperm quality in adult males. To maintain the quality of this meta-analysis, articles will be excluded when they are reviews or duplicates of previous publication; there are no clear definitions of the study population, air pollutants, criteria for semen analysis, and the outcome assessment; (3) there are no sufficient data for meta-analyses.

Two independent investigators will participate in the screening process. If there are any uncertain data; a third reviewer will reassess the data and organize a discussion to solve the problems.

### 2.2 Data extraction

Relative data in participants under higher exposure and lower exposure to air pollution will be extracted by 2 investigators independently. Only participants under the highest and the lowest exposure will be enrolled if the original study contained more than 2 exposure levels. All data will be recorded in a standardized form and the following basic characteristics from each study will be extracted: first author's name, year of publication, specific outdoor air pollutants, period of the research, study design, country, ethnicity, age, group assignment, and sample size. The primary outcomes will be extracted as follows: semen volume, sperm concentration, total sperm count, progressive motility, total motility, normal morphology rate. The second outcomes will be extracted as follows: DNA fragmentation index and computer-aided semen analysis system measures including linearity of sperm motion, sperm curvilinear velocity, and sperm linear velocity.

### 2.3 Quality evaluation

The quality of the enrolled studies will be evaluated by Newcastle-Ottawa Scale star system (range, 0–9 stars), which focuses on 3 broad perspectives: the selection of the study groups, the comparability of the groups, and the ascertainment of either the exposure or outcome of interest. The number of stars is positively associated with the quality of the study.

### 2.4 Trial sequential analysis

Trial sequential analyses (TSAs) will be performed to obtain a more comprehensive assessment of analyses by controlling the risk of random error.^[20,21]^ The TSA software (TSA, version 0.9; Copenhagen Trial Unit, Copenhagen, Denmark, 2011) will be used in the current study.

### 2.5 Bias assessment

Funnel plot will be implemented to detect the risk of publication bias if there are more than 10 studies qualified for analysis. Otherwise, Begg's test and Egger's test will be used.

### 2.6 Statistic methods

Continuous data will be presented as mean and standard deviation (SD). SD value will be transformed if only standard error or the 95% confidence interval (CI) is provided. If only median and range are available, mean and SD will be transformed according to a previously described forum: Mean≈Median; SD≈Norm IQR = (P_75_–P_25_)∗0.7413 (IQR: inter-quartile range, P_75_: 75th percentile, P_25_: 25th percentile).^[22]^ The Higgins I^2^ statistic and Cochrane Q test will be used to evaluate the heterogeneity between enrolled studies.^[23]^ A random-effect model (inverse variance method) will be used when the *P* value for heterogeneity is <.05 or I^2^ > 50%. Otherwise, a fixed-effect model (DerSimonian–Laird method) will be applied.^[24]^ Pooled estimates with corresponding 95% CIs will be calculated to assess the specific effects of outdoor air pollution in semen quality. Stata version 12 (StataCorp LP, College Station, TX) will be used for statistical analyses.

## 3 Discussion

Air pollution is a leading environmental health issue worldwide. Based on several animal researches, air pollution can influence sperm quality through impairment of sperm DNA, disorders of hypothalamic pituitary axis and testicular spermatogenesis, and oxidative stress. Though fundamental researches have demonstrated the negative effects of air pollution on semen parameters, epidemiologic studies demonstrated inconsistent results. Meta-analysis is a powerful tool which can provide more reliable results by pooling the results of single studies, especially in explaining controversial conclusions. Previous meta-analyses demonstrated a trend that air pollution can impair semen quality. However, the sample sizes were small and the results were nonsignificant. Recently, a large amount of clinical studies has demonstrated the negative impacts of air pollution on sperm quality. Hence, we intend to conduct a systematic review and meta-analysis to evaluate the role of outdoor air pollution in semen parameters and provide evidence for the clinical practitioners and health policy makers. Besides, TSA will be used to verify the results. There may be some potential limitations in this meta-analysis and systematic review. First, the contents of air pollutants and the cut-off points between lower exposure and higher exposure to air pollution may vary among the enrolled studies, which can increase the heterogeneity between studies and result in potential bias. Second, this meta-analysis only includes studies published in English, which may lead to selection bias.

## Author contributions

**Conceptualization:** Hongjun Li.

**Data curation:** Hongjun Li.

**Formal analysis:** Jianzhong Zhang.

**Funding acquisition:** Hongjun Li.

**Investigation:** Hongjun Li.

**Methodology:** Jianzhong Zhang, Zhonglin Cai.

**Project administration:** Hongjun Li.

**Software:** Bin Yang.

**Supervision:** Hongjun Li.

**Writing – original draft:** Jianzhong Zhang.

**Writing – review & editing:** Hongjun Li.

## References

[1] Kihal-Talantikite W Legendre P Le Nouveau P Premature Adult Death and Equity Impact of a Reduction of NO (2), PM10, and PM2.5 Levels in Paris-A Health Impact Assessment Study Conducted at the Census Block Level. Int J Environ Res Public Health 2019;16:38. 10.3390/ijerph16010038 PMC6339124 30586915

[2] Tajudin M Khan MF Mahiyuddin WRW Risk of concentrations of major air pollutants on the prevalence of cardiovascular and respiratory diseases in urbanized area of Kuala Lumpur, Malaysia. Ecotoxicol Environ Saf 2019;171:290–300. 30612017 10.1016/j.ecoenv.2018.12.057

[3] Soleimani Z Darvishi Boloorani A Khalifeh R Short-term effects of ambient air pollution and cardiovascular events in Shiraz, Iran, 2009 to 2015. Environ Sci Pollut Res Int 2019;26:6359–67. 30617889 10.1007/s11356-018-3952-4

[4] Fu P Guo X Cheung FM The association between PM2.5 exposure and neurological disorders: a systematic review and meta-analysis. Sci Total Environ 2019;655:1240–8. 30577116 10.1016/j.scitotenv.2018.11.218

[5] Conforti A Mascia M Cioffi G Air pollution and female fertility: a systematic review of literature. Reprod Biol Endocrinol 2018;16:117. 30594197 10.1186/s12958-018-0433-z PMC6311303

[6] Lafuente R Garcia-Blaquez N Jacquemin B Outdoor air pollution and sperm quality. Fertil Steril 2016;106:880–96. 27565259 10.1016/j.fertnstert.2016.08.022

[7] Jurewicz J Dziewirska E Radwan M Air pollution from natural and anthropic sources and male fertility. Reprod Biol Endocrinol 2018;16:109. 30579357 10.1186/s12958-018-0430-2 PMC6304234

[8] Zhang J Yang B Cai Z The negative impact of higher body mass index on sperm quality and erectile function: a cross-sectional study among Chinese males of infertile couples. Am J Mens Health 2019;13:1557988318822572. 30602337 10.1177/1557988318822572 PMC6440062

[9] Bundhun PK Janoo G Bhurtu A Tobacco smoking and semen quality in infertile males: a systematic review and meta-analysis. BMC Public Health 2019;19:36. 30621647 10.1186/s12889-018-6319-3 PMC6325781

[10] Hardneck F Israel G Pool E Quantitative assessment of heavy metal effects on sperm function using computer-aided sperm analysis and cytotoxicity assays. Andrologia 2018;50:e13141. 30225848 10.1111/and.13141

[11] Ricci E Noli S Ferrari S Alcohol intake and semen variables: cross-sectional analysis of a prospective cohort study of men referring to an Italian Fertility Clinic. Andrology 2018;6:690–6. 30019500 10.1111/andr.12521

[12] Guven A Kayikci A Cam K Alterations in semen parameters of toll collectors working at motorways: does diesel exposure induce detrimental effects on semen? Andrologia 2008;40:346–51. 19032683 10.1111/j.1439-0272.2008.00867.x

[13] Calogero AE La Vignera S Condorelli RA Environmental car exhaust pollution damages human sperm chromatin and DNA. J Endocrinol Invest 2011;34:e139–43. 20959722 10.1007/BF03346722

[14] Wu L Jin L Shi T Association between ambient particulate matter exposure and semen quality in Wuhan, China. Environ Int 2017;98:219–28. 27866723 10.1016/j.envint.2016.11.013

[15] Liu Y Zhou Y Ma J Inverse association between ambient sulfur dioxide exposure and semen quality in Wuhan, China. Environ Sci Technol 2017;51:12806–14. 28937752 10.1021/acs.est.7b03289 PMC5822717

[16] Selevan SG Borkovec L Slott VL Semen quality and reproductive health of young Czech men exposed to seasonal air pollution. Environ Health Perspect 2000;108:887–94. 11017895 10.1289/ehp.00108887 PMC2556931

[17] De Rosa M Zarrilli S Paesano L Traffic pollutants affect fertility in men. Hum Reprod 2003;18:1055–61. 12721184 10.1093/humrep/deg226

[18] Boggia B Carbone U Farinaro E Effects of working posture and exposure to traffic pollutants on sperm quality. J Endocrinol Invest 2009;32:430–4. 19794293 10.1007/BF03346481

[19] Rubes J Rybar R Prinosilova P Genetic polymorphisms influence the susceptibility of men to sperm DNA damage associated with exposure to air pollution. Mutat Res 2010;683:9–15. 19800896 10.1016/j.mrfmmm.2009.09.010

[20] Zhang J Li X Yang B Alpha-blockers with or without phosphodiesterase type 5 inhibitor for treatment of lower urinary tract symptoms secondary to benign prostatic hyperplasia: a systematic review and meta-analysis. World J Urol 2019;37:143–53. 29948047 10.1007/s00345-018-2370-z

[21] Zhang J Yang B Xiao W Effects of testosterone supplement treatment in hypogonadal adult males with T2DM: a meta-analysis and systematic review. World J Urol 2018;36:1315–26. 29511802 10.1007/s00345-018-2256-0

[22] Yang L Wang G Du Y Remote ischemic preconditioning reduces cardiac troponin I release in cardiac surgery: a meta-analysis. J Cardiothorac Vasc Anesth 2014;28:682–9. 24103716 10.1053/j.jvca.2013.05.035

[23] Higgins JP Thompson SG Deeks JJ Measuring inconsistency in meta-analyses. BMJ 2003;327:557–60. 12958120 10.1136/bmj.327.7414.557 PMC192859

[24] DerSimonian R Laird N Meta-analysis in clinical trials. Control Clin Trials 1986;7:177–88. 3802833 10.1016/0197-2456(86)90046-2

